# A 3 Year Follow-Up After Discharge From Early Intervention Service- Are Patients Less Likely to Be in Contact With Mental Health Services After Discharge From Eis and Are They Healthier?

**DOI:** 10.1192/bjo.2022.381

**Published:** 2022-06-20

**Authors:** Giedre Cesnaite, Latha Weston

**Affiliations:** Barnet, Enfield and Haringey Mental Health NHS Trust, London, United Kingdom

## Abstract

**Aims:**

To determine the utilization of mental health (MH) services, antipsychotic use and weight gain 3 years after discharge from an Early Intervention Service (EIS).

**Methods:**

A retrospective anonymized survey was conducted of the trust electronic records of patients discharged from Barnet EIS in April to May 2018. 25 case records were identified of which 4 were excluded due to relocation out of area. Information was reviewed from the time of referral to 3 years post discharge. Data included patient demographics, number of referrals to home treatment team (HTT), inpatient admissions, medication and cardiometabolic parameters (weight and HbA1c) during this period.

**Results:**

21 records were analysed - 13 males, 3 females, average age of 24 years. 12 patients had been discharged to primary care of which 5 were re-referred to community mental health team (CMHT) during the 3 year follow-up. 9 patients were discharged to the CMHT of which 4 were later discharged to primary care.

There was no significant difference in the number of referrals to HTT and hospital admissions in the GP and CMHT follow-up groups (50% and 33%; 56% and 44% respectively). At the time of discharge from EIS 67% were on antipsychotic medication. At 3 year follow-up 90% in CMHT group continued antipsychotics. There was an average of 15.6 kg weight gain while under EIS with further 11.7 kg gain over the next 3 years under CMHT care. According to available data for those still in contact with MH services, no patients newly met criteria for pre-diabetes or diabetes. No records were available on our system pertaining to GP discharges.

**Conclusion:**

We discuss the impact of EIS on affecting future MH service contact. There were a similar number of future MH referrals regardless of initial discharge destination. We consider whether there may be a different quality of these contacts that need further inspection.

The majority discharged continued taking antipsychotic medication and experienced considerable weight gain. This significant ongoing weight gain in a group of young people only starting to use MH services is of great concern due to negative cardiometabolic impact. It highlights the need for urgent proactive attention to ensure the best physical and mental health for these patients.

